# Drosophila Free-Running Rhythms Require Intercellular Communication

**DOI:** 10.1371/journal.pbio.0000013

**Published:** 2003-09-15

**Authors:** Ying Peng, Dan Stoleru, Joel D Levine, Jeffrey C Hall, Michael Rosbash

**Affiliations:** **1**Department of Biology, Brandeis UniversityWaltham, MassachusettsUnited States of America; **2**Howard Hughes Medical Institute, Brandeis UniversityWaltham, MassachusettsUnited States of America

## Abstract

Robust self-sustained oscillations are a ubiquitous characteristic of circadian rhythms. These include Drosophila locomotor activity rhythms, which persist for weeks in constant darkness (DD). Yet the molecular oscillations that underlie circadian rhythms damp rapidly in many Drosophila tissues. Although much progress has been made in understanding the biochemical and cellular basis of circadian rhythms, the mechanisms that underlie the differences between damped and self-sustaining oscillations remain largely unknown. A small cluster of neurons in adult Drosophila brain, the ventral lateral neurons (LN_v_s), is essential for self-sustained behavioral rhythms and has been proposed to be the primary pacemaker for locomotor activity rhythms. With an LN_v_-specific driver, we restricted functional clocks to these neurons and showed that they are not sufficient to drive circadian locomotor activity rhythms. Also contrary to expectation, we found that all brain clock neurons manifest robust circadian oscillations of *timeless* and *cryptochrome* RNA for many days in DD. This persistent molecular rhythm requires pigment-dispersing factor (PDF), an LN_v_-specific neuropeptide, because the molecular oscillations are gradually lost when *Pdf^01^* mutant flies are exposed to free-running conditions. This observation precisely parallels the previously reported effect on behavioral rhythms of the *Pdf^01^* mutant. PDF is likely to affect some clock neurons directly, since the peptide appears to bind to the surface of many clock neurons, including the LN_v_s themselves. We showed that the brain circadian clock in Drosophila is clearly distinguishable from the eyes and other rapidly damping peripheral tissues, as it sustains robust molecular oscillations in DD. At the same time, different clock neurons are likely to work cooperatively within the brain, because the LN_v_s alone are insufficient to support the circadian program. Based on the damping results with *Pdf^01^* mutant flies, we propose that LN_v_s, and specifically the PDF neuropeptide that it synthesizes, are important in coordinating a circadian cellular network within the brain. The cooperative function of this network appears to be necessary for maintaining robust molecular oscillations in DD and is the basis of sustained circadian locomotor activity rhythms.

## Abstract 2

Circadian rhythms are characterized by robust molecular oscillations, which are shown here to require a brain region-specific neuropeptide, PDF, for maintenance and coordination

## Introduction

Circadian rhythms of diverse organisms are based on similar intracellular molecular feedback loops (Dunlap 1999; Allada et al. 2001; Panda et al. 2002). Based on this view, it is believed that one or a small number of clock cells are sufficient for self-sustained rhythms (Dunlap 1999). This is despite the complex cellular organizations of many tissues, organisms, and systems (Kaneko and Hall 2000; Schibler and Sassone-Corsi 2002).

In Drosophila, circadian clocks have been identified in a diverse range of cell types throughout the head and the body (Glossop and Hardin 2002; Hall 2003). However, the clocks in different cells are considered nonidentical (Krishnan et al. 2001; Glossop and Hardin 2002; Levine et al. 2002a; Schibler and Sassone-Corsi 2002). In many tissues, molecular oscillations undergo rapid damping without environmental timing cues (Hardin 1994; Plautz et al. 1997; Stanewsky et al. 1997; Giebultowicz et al. 2000). This is similar to the damping of in vitro rhythms in some mammalian tissues (Balsalobre et al. 1998; Schibler and Sassone-Corsi 2002). In contrast, the Drosophila “core pacemaker” is believed to maintain robust oscillations for a long time in constant darkness (DD) with little or no damping, such that circadian behaviors can persist under such conditions (Dowse et al. 1987). Indeed, self-sustaining oscillations are a defining characteristic of true circadian rhythms and are believed to be required of a fully functional rhythmic cell. The differences between the “core pacemaker” and the clock machinery within damping cells or systems are unknown.

The six clusters of approximately 100 clock neurons in the adult Drosophila brain are well characterized (Kaneko and Hall 2000). Recent studies have focused principally on one of these groups, the small ventral lateral neurons (s-LN_v_s), as the best “core pacemaker” candidate for the following reasons: (1) in the developmental mutant *disco*, the presence of LN_v_s correlates with the maintenance of behavior rhythmicity (Helfrich-Förster 1997); (2) LN_v_s specifically express the neuropeptide pigment-dispersing factor (PDF), and the *Pdf^01^*-null mutant loses behavioral rhythmicity under DD conditions (Renn et al. 1999); (3) genetic ablation of the LN_v_s by expressing proapoptotic genes causes the loss of rhythmicity in DD (Renn et al. 1999); and (4) the s-LN_v_s maintain robust molecular oscillations for at least for 2 days in DD (Yang and Sehgal 2001; Shafer et al. 2002), in contrast to at least some other brain neurons and nonneuronal tissues. This final property suggests that these cells might fulfill the self-sustaining criterion for the “core pacemaker.” Indeed, the s-LN_v_s have been proposed to the primary pacemaker cells that generate locomotor activity rhythms (Helfrich-Förster 1997; Renn et al. 1999; Emery et al. 2000). Consistent with this cell-autonomous view of circadian rhythmicity, it has been shown that the LN_v_s possess all components of a fully functional, independent circadian clock: the photoreceptor cryptochrome, the rhythm-generating feedback loops, and a putative output factor, the neuropeptide PDF (Emery et al. 2000). Our pursuit of the self-sustaining “core pacemaker” of the Drosophila circadian system began with a test of the s-LN_v_ cell-autonomous clock hypothesis.

## Results

### LN_v_s Cannot Support Circadian Behavior Independently

**Figure 1:**
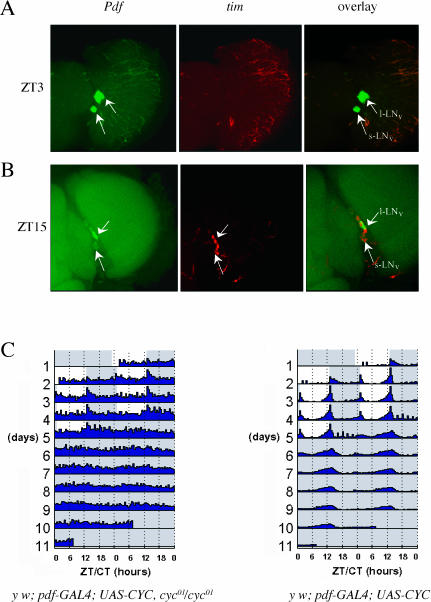
Rescuing Molecular Oscillations within the LN_v_s Is Not Sufficient to Rescue Locomotor Activity Rhythms The rescued mutant genotype is *y w*;*pdf–GAL4;UAS–CYC*,*cyc^01^*/*cyc^01^*. The flies were entrained in standard LD conditions and timepoints taken. Molecular oscillations were examined by whole-mount in situ hybridization of the *tim* gene. Double staining with a *Pdf* probe was used to label the LN_v_s neuronal group. (A and B) These show representative duplicate experiments. No *tim* mRNA signal is detectable in the dorsal region of the brain. The lower arrows point to the s-LN_v_s and the upper arrows to the l-LN_v_s. (A) Brain taken at timepoint ZT3. Panels shown from left to right are *Pdf* (green, FITC labeled), *tim* (red, Cy3 labeled), and an image overlay. (B) Brain taken at timepoint ZT15. Panels shown from left to right are *Pdf* (green, FITC labeled), *tim* (red, Cy3 labeled), and an image overlay. (C) The double-plotted actograms of rescue mutant and control flies in a standard LD:DD behavior assay. The colors on the background indicate the lighting conditions of the behavior monitors (white, lights on; light blue, lights off). In the actogram, the average locomotor activity of the group of flies is plotted as a function of time. The left panel shows the actogram of the rescued mutant flies (*y w;pdf–GAL4/+;UAS–CYC,cyc^01^*/*cyc^01^*, n = 30). RI (rhythm index; Levine et al. 2002a) = 0.14. The right panel shows the actogram for the rescued wild-type (control) flies (*y w;pdf–GAL4/+;UAS–CYC/+*, n = 32, RI = 0.61).

To test whether the LN_v_s can support free-running circadian locomotor activity rhythms independently of other functional clock cells, we restricted pacemaker activity to these few PDF-expressing cells. CYCLE (CYC) is a bHLH–PAS protein (Rutila et al. 1998) and forms a heterodimeric transcription factor with CLOCK (CLK), another bHLH–PAS protein (Allada et al. 1998). CYC is an essential component of the Drosophila circadian oscillator transcriptional feedback loop (Glossop et al. 1999). The *cyc^01^* nonsense mutation completely eliminates molecular oscillations, and the direct target genes *period (per)* and *timeless (tim)* mRNAs are essentially undetectable (Rutila et al. 1998). Behavioral rhythms are also absent in the *cyc^01^* homozygous mutant strain (Rutila et al. 1998). We rescued *cyc^01^* specifically in the LN_v_s, by using a well-characterized *pdf–GAL4* driver (Renn et al. 1999) in combination with a *UAS–CYC* transgene to express ectopically wild-type CYC. Since CYC is apparently not a rate-limiting component of active dCLK–CYC complexes (Bae et al. 2000) and does not undergo molecular oscillations itself (Rutila et al. 1998), we expected that CYC overexpression would not cause circadian oscillator dysfunction. Indeed, the presence of the two transgenes did not affect locomotor activity rhythms in a wild-type background (Figure 1C, right panel).

The rescued mutant flies (*pdf–GAL4;UAS–CYC,cyc^01^/cyc^01^*) were examined by two independent criteria. First, molecular oscillations were assayed by in situ hybridization with a *tim* probe (Figure 1A and 1B). *tim* RNA levels undergo robust cycling in wild-type flies, with a trough at ZT3 and a peak at ZT15 (Sehgal et al. 1994). This is also true within all individual clock neurons (Zhao et al. 2003). *tim* mRNA cycled in the LN_v_s (Figure 1A and B), indicating successful rescue of the molecular oscillator within these cells. The fact that other clock neurons were still *tim* mRNA-negative (Figure 1A and B) suggests that CYC and the rest of the molecular machinery can function cell autonomously, at least in the LN_v_s under these light–dark (LD) conditions. The observed oscillations are also not passively driven by light, since they persisted in DD, at least in the s-LN_v_s (Figure S1). Second, locomotor activity rhythms were examined by standard behavioral criteria. The transgenic flies were completely arrhythmic in DD. They were also arrhythmic under LD conditions, as the flies failed to anticipate the discontinuous transitions from light to dark or from dark to light (see Figure 1C, left panel; Rutila et al. 1998). In summary, the behavioral phenotypes were indistinguishable from those of the parental *cyc^01^* mutant strain.

### Brain Clock Neurons Manifest Robust Molecular Oscillations in DD

**Figure 2:**
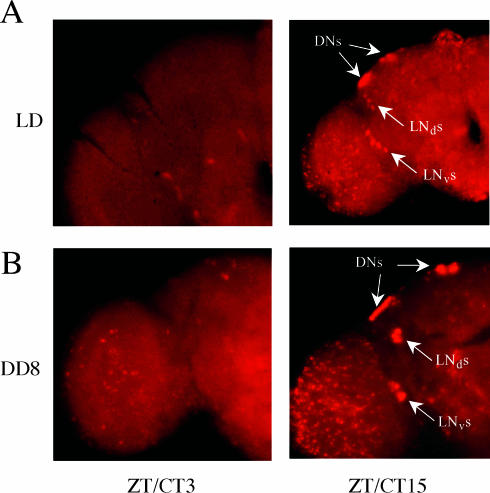
All Brain Clock Neuronal Groups Maintain Robust Oscillations of *tim* RNA Levels in DD Wild-type flies were entrained for at least 3 days and then released into DD. *tim* RNA was assayed at trough (left panels) and peak (right panels) timepoints by whole-mount in situ hybridization. Wild-type flies in LD (A) were compared with the eighth day of DD (B). On the eighth day of DD, the locomotor activities of the fly population were still in close synchrony, without any obvious phase spreading (data not shown). Left panels, brains at ZT3 (A) or CT3 (B); right panels, brains from ZT15 (A) or CT15 (B). Both (A) and (B) are representative of three replicate experiments.

The insufficiency of LN_v_ molecular rhythmicity indicates that one or more additional groups of rhythmic clock neurons are required for behavioral rhythmicity. We considered that robust molecular cycling under extended constant darkness conditions might be a good criterion for identifying these cell groups, because prior biochemical studies showed that some head and brain locations undergo damping of molecular oscillations under free-running conditions (Hardin 1994; Stanewsky et al. 1997). This conclusion has been extended by more recent immunohistochemical observations (Yang and Sehgal 2001; Shafer et al. 2002). The criterion of maintaining persistent and robust molecular rhythms in DD therefore suggests that only a limited set of brain locations are likely to be free-running pacemaker candidates. In order to identify these neurons, we assayed fly brains by *tim* in situ hybridization after 8 days in DD. To our surprise, we found that all *tim-*expressing brain cell groups (including both large ventral lateral neurons [l-LN_v_s] and small ventral lateral neurons [s-LN_v_s], doral lateral neurons [LN_d_s], and all three groups of dorsal neurons [DNs]) still cycle robustly at this time (Figure 2). Previous studies have reported that the l-LN_v_s fail to maintain oscillations at the beginning of DD (Yang and Sehgal 2001; Shafer et al. 2002). We have reproduced these observations, but noticed that the l-LN_v_s “adapt” to constant conditions by becoming rhythmic once again after about 2 days in DD (data not shown). These results clearly distinguish the brain from the eyes and other peripheral tissues, which rapidly lose coherent molecular oscillations under free-running conditions (Hardin 1994; Plautz et al. 1997; Stanewsky et al. 1997; Giebultowicz et al. 2000). Although this approach failed to identify the additional neuronal groups necessary for behavioral rhythms, it suggests that many of these brain neuronal groups might act together in a network to support robust rhythms.

### Sustained Molecular Oscillation in Constant Darkness Requires PDF

This association between robust molecular oscillations in all brain clock cells and behavioral rhythms in DD also made us consider the role of the neuropeptide PDF. The *Pdf^01^* mutant strain is unique among identified Drosophila circadian mutants, as it has little effect under LD conditions, but loses behavioral rhythmicity gradually and specifically in DD (Renn et al. 1999). This phenotype might reflect a disassociation between behavioral rhythmicity and the underlying molecular oscillations, as predicted from the role of PDF as a circadian output signal; it is proposed to connect the molecular oscillation in the LN_v_s to locomotor activity (Renn et al. 1999).

We considered a completely different interpretation, namely, that PDF contributes to the functional integration of several brain clock neuronal groups, which is necessary to sustain molecular as well as behavioral rhythmicity under constant conditions. This fits well with previous studies of PDF in other organisms (Rao and Riehm 1993; Petri and Stengl 1997). In contrast to the canonical output model, this possibility suggests that the *Pdf^01^* mutant might manifest unusual molecular oscillations within clock neurons, especially under DD conditions. To address this issue experimentally, we examined *Pdf^01^* mutant flies by *tim* in situ hybridization.

**Figure 3:**
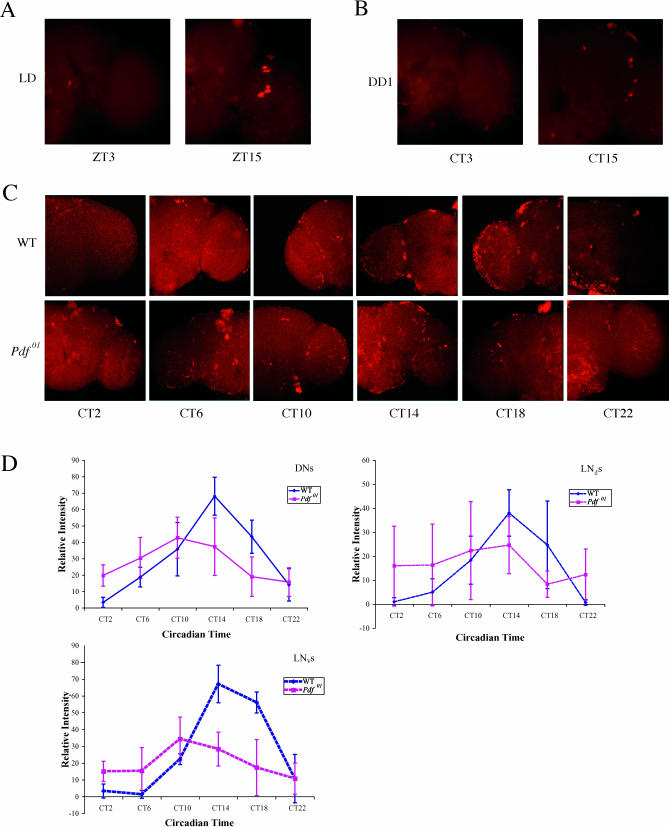
Molecular Oscillations of *tim* RNA Damp in DD in the *Pdf^01^* Mutant *tim* RNA oscillations were examined in the *Pdf^01^* mutant under both LD (A) and different days in DD ([B] and [C]), by whole-mount in situ hybridization. (A), (B), and (C) are representative images from replicas of three experiments. (A) The left panel is from ZT3, and the right panel is from ZT15. A normal *tim* oscillation profile is observed compared to that of wild-type (see Figure 2A). (B) Brains from the *Pdf^01^* mutant in the first day of DD. Left panel, CT3; right panel, CT15. Oscillations are comparable to those in LD. (C) Brains taken in the fourth day of DD. Six timepoints were taken throughout the circadian day. The sequence of panels from left to right is CT2, 6, 10, 14, 18, and 20, respectively. Wild-type brains (top row) were assayed in parallel with those from the *Pdf^01^* mutant (bottom row). See text for details. (D) Quantification of (C). Relative intensities are taken from normalized mean pixel intensities. Different clock neuronal groups were quantified independently and compared between wild-type (blue curves) and *Pdf^01^* mutant (purple curves). The panels from left to right are quantification of *tim* RNA oscillation in the DNs, in the LN_d_s, and in the LN_v_s. Reduced cycling amplitude and a significant advanced phase were observed in the fourth day of DD. See text for details.

In *Pdf^01^* flies, all clock neurons had robust *tim* RNA oscillations in LD, and the cycling phase and amplitude were comparable to those of wide-type flies (Figure 3A). The mutant flies were then released into DD and assayed at various times thereafter. In the first day of DD, cycling was similar to that observed in LD (Figure 3B). By the fourth day of DD, however, the cycling amplitude was much reduced in all clock neurons (Figure 3C and 3D). This was most evident from the unusually high signal in the CT2 sample; in wild-type flies, no *tim* signal was detected in any clock neuron at this timepoint (Figure 3C, left panels). There was also a reduced signal strength at the peak time, CT14 (Figure 3C, fourth panel from the left). The result parallels the damping of behavioral rhythms in the *Pdf^01^* mutant strain (Renn et al. 1999).

Despite the gradual fading of locomotor activity rhythms in DD, a significant fraction of *Pdf^01^* mutant flies is still weakly rhythmic after 4 d of DD (Renn et al. 1999). By tracking their locomotor activity phases, we observed that most of them had accumulated an approximately 4-hour phase advance relative to wild-type flies by the fourth day in DD. This is consistent with the measured ca. 23-hour periods of these weakly rhythmic flies (1-hour phase advanced per day for 4 days) as well as their advanced evening activity peak in LD (Renn et al. 1999). Quantitation of the *tim* in situ hybridization signal showed that there was a comparable one-point (4 h) advance in the peak of *tim* RNA and also confirmed the reduced cycling amplitude (Figure 3D). In order to eliminate the possibility that the observed damping is caused by the asynchrony of the *Pdf^01^* fly population, locomotor activities were tracked in real time. Individual flies were then removed from the monitors to assay *tim* RNA levels. Identical damped molecular oscillations were also observed in this case (data not shown). Taken together, the results indicate an excellent quantitative correspondence in phase and amplitude between the *tim* RNA rhythms and the behavioral rhythms in all clock neurons of the *Pdf^01^* strain.

**Figure 4:**
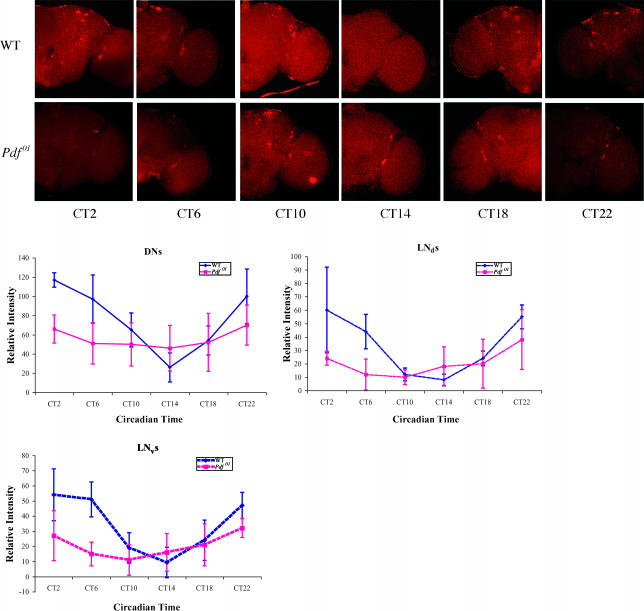
*cry* RNA Oscillation Amplitude Is Also Reduced by the Fourth Day of DD in the *Pdf^01^* Mutant *cry* RNA expression in the brain was examined at the fourth day of DD by whole-mount in situ hybridization using a *cry* probe. Timepoints were taken every 4 hours throughout the circadian day. The sequence of panels from left to right is CT2, 6, 10, 14, 18, and 20, respectively. Wild-type brains (top row) were analyzed in parallel with those from the *Pdf^01^* mutant (bottom row). Shown are representative images from duplicate experiments. Quantification of *cry* RNA oscillations in different cell groups is as shown in Figure 3. Ubiquitous damping of the cycling amplitude in the different cell groups was observed in the *Pdf^01^* mutant.

To extend these observations, we also assayed *cryptochrome (cry)* mRNA oscillations by in situ hybridization. *cry* is expressed in a similar clock neuron pattern to *tim*, but it has a peak expression at ZT2 and a trough at ZT14 (Emery et al. 1998; Zhao et al. 2003). This phase is opposite to that of *tim* and other CLK–CYC direct target genes and reflects the fact that *cry* is only indirectly regulated by this heterodimeric transcription factor; CLK–CYC directly regulates the transcription factors PDP1 and VRILLE, which then regulate *cry* (Cyran et al. 2003; Glossop et al. 2003). Despite these differences between *tim* and *cry*, a similar result was obtained for *cry* in the *Pdf^01^* strain in the fourth day of DD (Figure 4), i.e., a reduced cycling amplitude compared to the fourth day of DD in a wild-type strain. This is suggested by the in situ pictures and is strongly indicated by the quantitation (Figure 4). The correspondence between the *tim* and *cry* mRNA patterns indicates that the entire circadian transcriptional program damps in the mutant strain in DD, which underlies the behavioral damping.

### PDF Is Likely to Act upon Clock Neurons Directly

It is noteworthy that the mRNA oscillations damp uniformly in the *Pdf^01^* mutant strain, including the PDF-expressing LN_v_s (see Figures 3 and 4). Since PDF is a neuropeptide (Rao and Riehm 1993), it is unlikely to exert a direct intracellular effect on the LN_v_ transcriptional machinery. A more conservative interpretation is that PDF maintains intercellular communication between individual LN_v_ neurons (Petri and Stengl 1997) and/or between the LN_v_s and other cells; the communication is essential for self-sustained molecular rhythms within the LN_v_s. Although this “feedback” could be quite indirect, the l-LN_v_s project to the contralateral LN_v_s through the posterior optic tract. Moreover, the s-LN_v_s project dorsally to the superior protocerebrum, the location of the DNs. (Helfrich-Förster 1995). These anatomic features suggest that PDF might bind directly to clock neurons.

**Figure 5:**
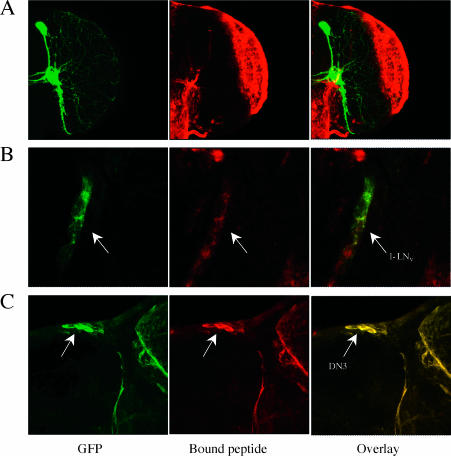
A PDF Peptide Binds to Many Cells, Including Several Clock Neuronal Groups In vitro biontinylated PDF peptide was used to visualize the peptide binding locations (middle panels, with Cy3) in the brain (see Materials and Methods for details). We used membrane-bound GFP (green panels on the left) to label specific circadian neurons as well as their projections (right panels show the overlay of both channels). (A) The brain is from flies with labeled LN_v_s *(y w,UAS–mCD8iGFP;pdf–GAL4)*. Numerous cells at the periphery of the medulla have the vast majority of the bound PDF peptide signal within the brain. This region receives widespread dendritic arborizations from the l-LN_v_s. (B) Bound PDF peptide was also detected on the surface of LN_v_s at a lower intensity. LN_v_ cell bodies were labeled using *UAS–mCD8iGFP;pdf–GAL4*. Since the signal from the Cy3 channel was much weaker than the GFP signal, we reduced the output gain from the GFP channel. Sequential scanning was used to prevent cross-talk between the two channels. (C) *y w,UAS–mCD8iGFP;tim–GAL4/+* flies were used to label all circadian neurons. In the dorsal region shown in this series, the arrow points to a group of DN3 neurons.

To test this hypothesis, in vitro biotinylated PDF peptide was incubated with fixed adult brains under near physiological conditions. The bound peptide was then detected in situ with a streptavidin-conjugated enzymatic amplification reaction. The vast majority of the signal localized with numerous cells at the periphery of medulla (Figure 5A). This is exactly where the l-LN_v_s send large arborizations as their centrifugal projections (Helfrich-Förster 1995). Importantly, signal was also detected coincident with the LN_v_s (Figure 5B) and likely DN3 clock neurons (Figure 5C) within the superior protocerebrum region, i.e., the bound peptide colocalized with GFP when the brains were from a strain with GFP-labeled clock neurons. Staining intensity was temporally constant; i.e., there was no systematic variation in signal intensity with circadian time. Although we obtained identical results with two differently biotinylated PDF peptides and there was no staining with two other biotinylated control peptides, we had difficulty to compete specifically the signal with nonbiotinylated PDF (see Materials and Methods). Moreover, PDF peptide staining of clock neurons was not reliably detected in every brain, in contrast to optic lobe staining. Nonetheless, we never detected peptide staining of other neurons in the vicinity of the LN_v_s; i.e., signal in this region of the brain was always coincident with the GFP-labeled LN_v_s. The peptide staining therefore suggests that PDF acts on the LN_v_s in an autocrine or paracrine fashion as well as on other clock neurons, but the results do not exclude additional, more indirect modes of action.

## Discussion

The strong behavioral phenotype of the *Pdf^01^* mutant strain in DD indicates that PDF makes an important contribution to free-running circadian rhythms. It was, however, unanticipated that the *Pdf^01^* mutant would have an additional effect on transcriptional oscillations within most if not all clock neurons. This observation extends the tight parallel between strong behavioral rhythms and robust transcriptional rhythms and suggests that the behavioral damping is due to the transcriptional damping (Marrus et al. 1996). In contrast to this strong effect of the *Pdf^01^* mutation on free-running rhythms, the molecular as well as behavioral rhythms of these mutant flies are nearly normal under LD conditions. We now interpret this difference to indicate that intercellular communication among different clock cells and neuronal groups is less important when they can independently receive photic information via cryptochrome. This probably serves not only to synchronize clock neurons but also to reinforce and strengthen the molecular oscillation (Emery et al. 1998; Stanewsky et al. 1998).

The damping phenotype includes the LN_v_s, which have been proposed to be the principal pacemaker neurons in Drosophila (Helfrich-Förster 1997; Renn et al. 1999). Their counterparts in mammals, the suprachiasmatic nucleus (SCN) neurons, can support circadian rhythms independently (e.g., Sujino et al. 2003). However, our data indicate that the LN_v_s cannot support locomotor activity rhythms without other clock cell groups (see Figure 1). A similar attempt to rescue behavioral rhythms of an arrhythmic *Clk* mutant also failed (Allada et al. 2003). Although the negative result shown here might be due to developmental defects of the *cyc^01^* mutation (Park et al. 2000), the conclusion fits well with a role for PDF in functional cooperation between individual neuronal groups. Indeed, it appears that PDF secretion comprises much of what the LN_v_s contribute to rhythms, as the phenotype of flies missing the LN_v_s is virtually identical to that of the *Pdf^01^* strain (Renn et al. 1999). There is less known about the roles of other clock neurons, although they do have specific wiring properties (Kaneko and Hall 2000) as well as specific sets of gene expression profiles (unpublished data). An additional indication that other clock neurons contribute to locomotor activity rhythms is that LD behavioral rhythms do not require the LN_v_s (Hardin et al. 1992; Renn et al. 1999). As the *Pdf^01^* strain also has a strong effect on geotaxis (Toma et al. 2002), clock neurons may even contribute to other behavioral modalities.

The staining pattern suggests that the PDF ligand contacts a receptor on the surface of clock neurons, including the LN_v_s themselves. This is consistent with the notion that PDF acts as an important intercellular cell communication molecule within the Drosophila circadian system. The dorsal projections of the s-LN_v_s stain rhythmically with anti-PDF antibodies, and it has been suggested that released PDF affects dorsal clock neurons (Helfrich-Förster et al. 2000). Indeed, ectopic expression of PDF in neurons that project to the dorsal brain region causes severe rhythm defects, suggesting that misregulation of this signaling causes circadian system dysfunction (Helfrich-Förster et al. 2000). Our staining with a PDF peptide indicates that the PDF signaling to the DNs may be direct. Although rhythmic PDF staining is restricted to the s-LN_v_ terminals (Park et al. 2000), this could be because a smaller fraction of PDF is released from the l-LN_v_ terminals. Some of these processes follow the posterior optic track to the opposite side of the brain. Taken together with the LN_v_ peptide staining, it is likely that PDF from the l-LN_v_s signals contralaterally and positively influences clock cells on the opposite side of the brain. A very recent study of the Drosophila prothoracic gland (PG) clock and eclosion rhythms suggests that the LN_v_s also control the PG clock via PDF signaling (Myers et al. 2003). This raises the possibility that PDF not only synchronizes brain clock neurons, but also keeps peripheral clocks in pace with the core brain network.

The *Pdf^01^* molecular phenotype implies that the wild-type organization of the system normally supports the individual clock cells as well as the entire circadian program in DD. Although we do not know that all molecular aspects of rhythms damp in DD in *Pdf^01^* flies, we suggest that damped transcriptional rhythms are the intracellular default state in Drosophila and are manifest without the driving and entraining LD cycle or without a functionally integrated clock network. This view is also consistent with recent studies showing that electrical silencing of clock neurons eliminates free-running molecular as well as behavioral rhythms (Nitabach et al. 2002). It will be interesting to learn how PDF signaling connects to the intracellular transcriptional machinery.

We note that communication among clock neurons is likely to be important in other organisms. The ability of PDF to phase-shift the cockroach circadian clock (Petri and Stengl 1997) is more consistent with our proposal than with a simple role in clock output. A recent study of VPAC(2) receptor knock-out mice (Harmar et al. 2002) showed that these mice fail to sustain behavioral rhythms and have molecular rhythms defects within the SCN. This raises the intriguing possibility that SCN neurons as well as Drosophila clock neurons may require network integration to sustain free-running intracellular oscillations.

## Materials and Methods

### 

#### Drosophila genetics.

Full-length *cyc* cDNA was obtained from BDGP cDNA clone GM02625 and was tagged with hemagglutinin (HA) epitope by PCR cloning. CYC–HA was subsequently cloned into pUAST to generate pUAS–CYC–HA. The transformation plasmid was used to generate transgenetic flies. A third chromosome insertion line *(UAS–CYC–HA15)* was used subsequently. All wild-type flies and specimens were taken from a Canton-S stock.

The circadian driver lines *pdf–GAL4* (Renn et al. 1999), *tim–GAL4* (Kaneko and Hall 2000), as well as the *cyc^01^* (Rutila et al. 1998) and *Pdf^01^* (Renn et al. 1999) mutant strains have been previously described. All molecular and behavioral analyses were conducted on flies entrained at 25°C.

#### GFP expression analysis.

To visualize the axon projections from circadian neurons, a *UAS–mCD8GFP* line labeling the cell membrane was crossed with various circadian GAL4 drivers. The progeny brains were dissected in PBS and fixed in 3.7% paraformaldehyde in PEM. After rinses in PBS plus 0.3% Triton and PBS, brains were mounted in Vectashield mounting medium (Vector Laboratories, Burlingame, California, United States) and imaged on a Leica laser scanning confocal microscope. Optical sections were taken at 1–2 μm intervals and used to construct a maximum projection image for each brain.

#### In situ mRNA hybridization on adult brain whole mounts.

In situ hybridization of *tim* and *cry* was done as described previously (Zhao et al. 2003). The maximum projection images taken from a Leica laser scanning confocal microscope were used for the quantification. The quantification was done using three brain images per sample with Leica confocal software. The mean pixel intensities of cell groups were normalized by subtracting the average of two general background areas in the brain.

#### Behavioral analysis.

Flies were entrained for 3–5 d in 12 h light:12 h dark (LD) conditions before release into DD. Locomotor activities of individual flies were monitored using Trikinetics Drosophila Activity Monitors (Waltham, Massachusetts, United States). The analysis was done by using a signal processing toolbox (Levine et al. 2002b). Autocorrelation and spectral analysis were used to assess rhythmicity and to estimate the period. The phase information was extracted using circular statistics (Levine et al. 2002b). In some cases, the phases of individual *Pdf^01^* flies were also examined by inspection.

#### In vitro peptide binding assay.

Biotinylation of the PDF peptide was with EZ-Link Sulfo–NHS–LC–Biotin reagent (Pierce Biotechnology, Rockford, Illinois, United States), following the manufacturer's instruction. Excess biotinylation reagent was removed by prolonged incubation in Tris–HCl buffer (1 M [pH 7.5]) followed by protein purification through a Polyacrylamide 1800 desalting column (Pierce Biotechnology). A control neuropeptide, allatostatin I (Sigma-Aldrich, St. Louis, Missouri, United States), was biotinylated using the same method. A second control was a synthetic, biotinylated peptide derived from the Drosophila PER protein (a gift from P. Nawathean). In addition, a new N-terminus biotinylated PDF peptide was chemically synthesized de novo (Sigma-Aldrich). Identical results were obtained with the two PDF peptides, and no specific signal was obtained with the two control peptides.

To detect the binding of the neuropeptide in the CNS of Drosophila, brains were dissected in PBS and fixed in 3.7% paraformaldehyde in PEM for 30 min. After they were rinsed in PBS plus 0.3% Triton and blocked using 1% FBS or BSA, biotinylated peptide was incubated with the brains at a final concentration of 0.2 μg/ml. The brains were washed thoroughly with TNT (0.1 M Tris–HCl [pH 7.5], 0.15 M NaCl, 0.05% Tween 20). The bound peptide was subsequently detected through the biotin label using streptavidin–HRP (NEN LifeScience, now Perkin-Elmer, Torrance, California, United States) and fluorescent tyramides (NEN LifeScience). A detailed protocol is provided as Protocol S1. For the competition assay, unlabeled peptide was added at a 200- to 5000-fold concentration increase in the blocking step; subsequent steps were as described above.

## Supporting Information

Figure S1 Rescued Molecular Oscillations Persist during DD in the s-LN_v_s The “rescued” mutant *y w; pdf–GAL4;UAS–CYC*,*cyc^01^*/*cyc^01^* was released into DD after entrainment and assayed by *tim* whole-mount in situ hybridization on the fourth day of DD. A *Pdf* probe was used to label the LN_v_ group. Brains were taken at two opposite timepoints, CT3 (top panels) and CT15 (bottom panels). From left to right are *Pdf* (green, FITC labeled), *tim* (red, Cy3 labeled), and an image overlay. The lower arrows point to the s-LN_v_s and the upper arrows to l-LN_v_s. Whereas the l-LN_v_s show barely visible *tim* RNA oscillations under these conditions, the s-LN_v_s are obviously cycling. This difference suggests that the l-LN_v_s might damp more rapidly or be more light-dependent than the s-LN_v_s in this unusual genotype. (7.1 MB PDF).

Protocol S1 Short Protocol for Neuropeptide Biotinylation and Receptor Detection (23 KB DOC).

## Abbreviations

*clk*: *clock*
*cry*: *cryptochrome*
*cyc*: *cycle*
CT: circadian time
DD: constant darkness
DN: dorsal neuron
HA: hemagglutinin
LD: light–dark
l-LN_v_: large ventral lateral neuron
LN_d_: dorsal lateral neuron
LN_v_: ventral lateral neuron
PDF: pigment-dispersing factor
*per*: *period*
PG: prothoracic gland
SCN: suprachiasmatic nucleus
s-LN_v_: small ventral lateral neuron
*tim*: *timeless*
ZT: Zeitgeber time.
