# Supplementary figures and images for: Drosophila Free-Running Rhythms Require Intercellular Communication
Source: PLoS Biol. 2003 Sep 15;1(1):e13. doi: 10.1371/journal.pbio.0000013 (PMC193604; doi:10.1371/journal.pbio.0000013)

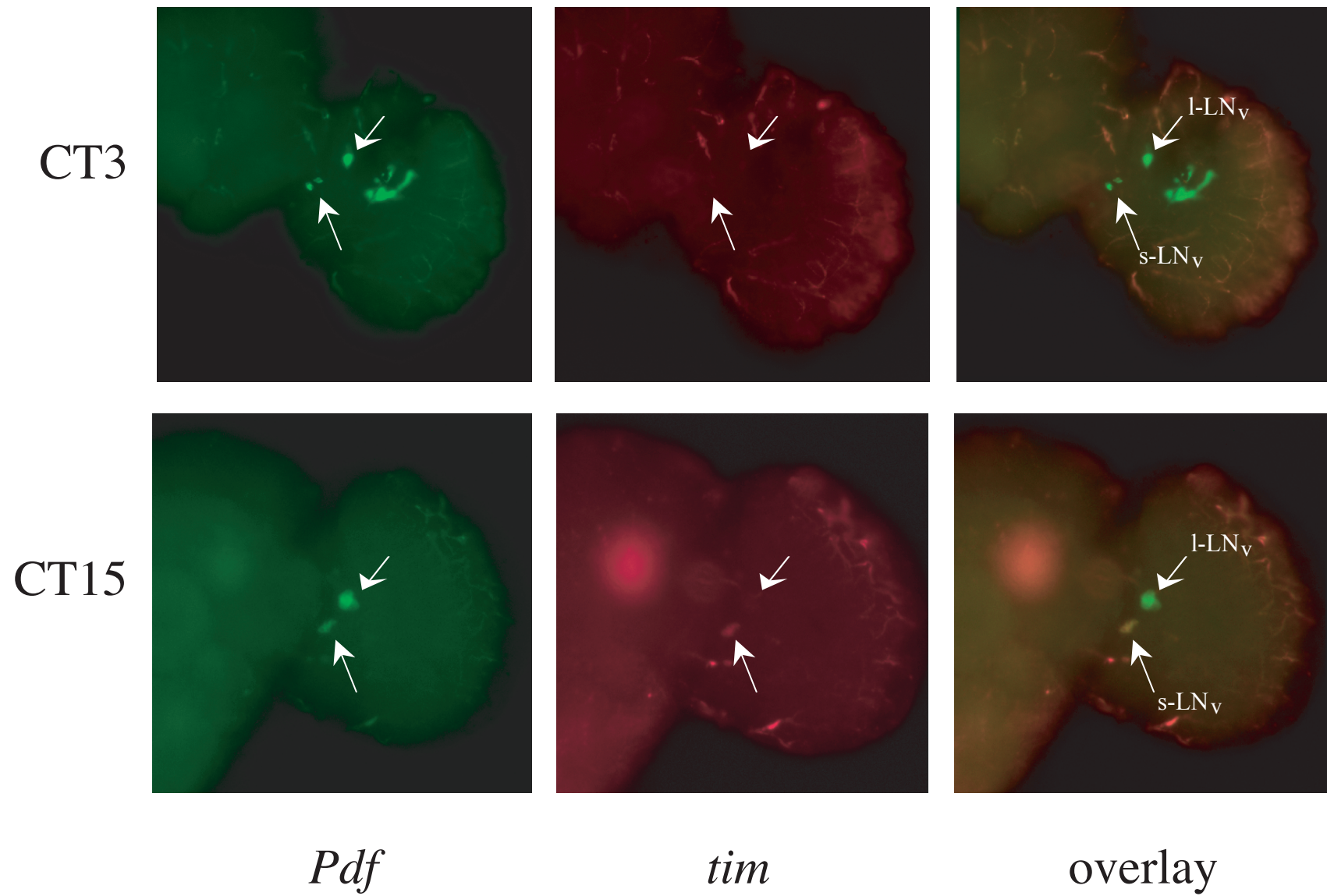

Figure S1. Peng et al.

Supplement: Figure S1 — The “rescued” mutant y w; pdf–GAL4;UAS–CYC,cyc01/cyc01 was released into DD after entrainment and assayed by tim whole-mount in situ hybridization on the fourth day of DD. A Pdf probe was used to label the LNv group. Brains were taken at two opposite timepoints, CT3 (top panels) and CT15 (bottom panels). From left to right are Pdf (green, FITC labeled), tim (red, Cy3 labeled), and an image overlay. The lower arrows point to the s-LNvs and the upper arrows to l-LNvs. Whereas the l-LNvs show barely visible tim RNA oscillations under these conditions, the s-LNvs are obviously cycling. This difference suggests that the l-LNvs might damp more rapidly or be more light-dependent than the s-LNvs in this unusual genotype. (7.1 MB PDF). [file pbio.0000013.sg001.pdf]
