# Supplementary material for: Drosophila Free-Running Rhythms Require Intercellular Communication
Source: PLoS Biol. 2003 Sep 15;1(1):e13. doi: 10.1371/journal.pbio.0000013 (PMC193604; doi:10.1371/journal.pbio.0000013)
Supplement: Protocol S1 — (23 KB DOC). [file pbio.0000013.sd001.doc]

Short protocol for neuropeptide biotinylation and receptor detection

Peptide labeling and purification:

1. weight ~1mg of peptide and dissolve into NH3 free PBS buffer, reaching the final concentration of 1mg/ml.
2. Based on the molecular weight of the peptide, determine the molar concentration of the 1mg/ml solution. Weight 20 time molar excess EZ-Link™ Sulfo-NHS-Biotin Reagents (pierce) in a separate tube.
3. Transfer the peptide solution in PBS into the reagent tube. Flip the tube to dissolve the reagent. Let it sit on ice for ~2 hrs.
4. Add equal volume of 2.0M Tris-cl (PH 7.5) in the tube to stop the reaction. Left on ice over night.
5. The labeled peptide can be used directly at this point, or go through desalting column to get rid of remaining active reagent (if the molecular weight of the peptide is less than 2KD, don’t desalt).
6. pre-equilibrate D-salt Polyacylamide 1800 desalting Gel (pierce) with 10 time bed volume of PBS. Apply the reaction mix on the top of the column, let it enter the gel completely. Collect elute while washing the column with cold PBS. The protein will get out with in the first 5ml elute.
7. Determine the protein concentration in each fraction, keep the peak fraction and discard the rest.
8. Aliquot the labeled protein and freeze for later usage.

Receptor detection:

1. Dissect the tissue of interest and fix appropriately. (for drosophila brain, we use 4% paraformadehyde in PEM buffer for 30 minutes)
2. Wash in PBT (PBS +0.1%tween-20) several times, in order to get rid of the remaining fix.
3. Block the tissue with appropriate blocking reagent (5% FBS, BSA, or et al., )
4. Dilute the labeled peptide with the blocking reagent, better to use a concentration gradient in the beginning. For PDF detection in drosophila brain, the best working concentration is around 20-200 ng/ml.
5. Incubate at 4˚C over night.
6. Wash with PBT thoroughly (about 10X at least).
7. Add streptavidin-HRP (NEN life science, 1:250 dilution in blocking buffer), room temperature for 2hr, or 4˚C over night.
8. Wash with TNT thoroughly.
9. Use the NEN TSA kit for the detection.
10. Wash with TNT thoroughly.
11. Mount the tissue.
